# Needle puncture fistulotomy: a new technique for needle-knife fistulotomy as a primary biliary access method for biliary cannulation

**DOI:** 10.1055/a-2248-0137

**Published:** 2024-02-15

**Authors:** Ahmed Sadek, Kazuo Hara, Nozomi Okuno, Shin Haba, Takamichi Kuwahara

**Affiliations:** 1 Internal Medicine Department, Faculty of Medicine, Helwan University, Cairo, Egypt; 2 Department of Gastroenterology, Aichi Cancer Center, Nagoya, Japan

Post-endoscopic retrograde cholangiopancreatography (ERCP) pancreatitis is a serious complication, occurring in 2%–10% of cases, with frequent cannulation attempts and precutting being the primary risk factors 1 .

Needle-knife fistulotomy, is an alternative method for accessing the common bile duct (CBD) without touching the papillary orifice and has been shown to reduce the risk of post-ERCP pancreatitis when used as an initial biliary access method 1 . We demonstrate our new technique of primary needle puncture fistulotomy (NPF) using an endoscopic submucosal dissection (ESD) needle-knife.

The major papilla is examined through measurement of the papillary roof from the mucosal fold to the top edge of the infundibulum (it should be greater than 5 mm). NPF is performed using a needle-knife (Splash M knife; Pentax, Tokyo, Japan). This knife has a metal plate at the needle base with a wider current conducting area, which was originally designed for marking the mucosa before ESD and provides a wide cutting diameter ( Fig. 1 **a** ). ERBE VIO 3 (Erbe Elektromedizin GmbH, Tübingen, Germany) is used with ENDO CUT I mode (effect 2, cut duration 2, cut interval 2).

**Fig. 1:**
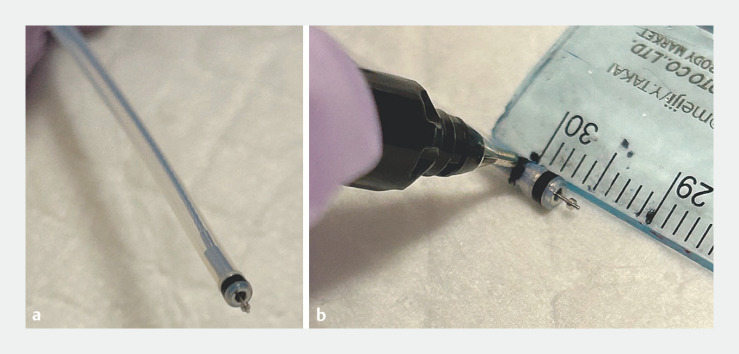
Marking the fistulotomy knife. **a** Splash M knife (Pentax, Tokyo, Japan), showing the needle tip and basal metal plate. **b** Black line mark made at 5 mm from the needle tip.

Before needle insertion, we mark the needle with a black line at 5 mm from the tip ( Fig. 1 **b** ). After determining the puncture point, the papilla is punctured until the 5 mm mark is reached or a special sign appears ( Video 1 ). Here, we describe the sign that indicates adequate depth of puncturing the papilla during NPF; we call it the “popping” sign.

Needle puncture fistulotomy: a new technique for primary needle knife fistulotomy.Video 1

While puncturing, we can see air bubbles or small fragments coming from the papillary orifice; at this point, we stop puncturing and proceed with CBD cannulation. Finally, biliary cannulation can be done. Extended sphincterotomy or balloon dilation can be performed depending on the indication of the procedure.

We believe that this technique is easier than conventional needle-knife fistulotomy. Further prospective studies are required to assess the safety and efficacy of this technique.

Endoscopy_UCTN_Code_TTT_1AR_2AC

## Notes

Endoscopy E-Videos https://eref.thieme.de/e-videos
*E-Videos* is an open access online section of the journal *Endoscopy* , reporting on interesting cases and new techniques in gastroenterological endoscopy. All papers include a high-quality video and are published with a Creative Commons CC-BY license. Endoscopy E-Videos qualify for HINARI discounts and waivers and eligibility is automatically checked during the submission process. We grant 100% waivers to articles whose corresponding authors are based in Group A countries and 50% waivers to those who are based in Group B countries as classified by Research4Life (see: https://www.research4life.org/access/eligibility/ ). This section has its own submission website at https://mc.manuscriptcentral.com/e-videos .
